# An Efficient and Sustainable Approach to Decarboxylative Cross-Coupling Using Silica Coated Magnetic Copper Nanocatalyst for the Synthesis of Internal Alkynes

**DOI:** 10.3389/fchem.2021.773855

**Published:** 2022-01-17

**Authors:** Manavi Yadav, Anju Srivastava, Rashmi Gaur, Radhika Gupta, Gunjan Arora, Rakesh Kumar Sharma

**Affiliations:** ^1^ Green Chemistry Network Centre, Department of Chemistry, University of Delhi, New Delhi, India; ^2^ Department of Chemistry, Hindu College, University of Delhi, Delhi, India

**Keywords:** magnetic, copper, nanocatalyst, decarboxylative cross-coupling, heterogeneous catalyst

## Abstract

A highly efficient magnetically separable copper nanocatalyst has been developed for decarboxylative cross-coupling reaction for the alkynylation of haloarenes using alkynoic acid as a reaction partner. The chemical nature, morphology, size, and magnetic properties of the prepared nanocatalyst were studied by SEM, TEM, EDS, FT-IR, VSM, and ICP techniques. Remarkably, this catalyst represents the first successful copper based heterogeneous system for this type of coupling that provides a low-cost, stable, and environmentally friendly magnetically recoverable entity that can be re-used for seven consecutive runs without appreciable loss in its catalytic performance.

**GRAPHICAL ABSTRACT:**
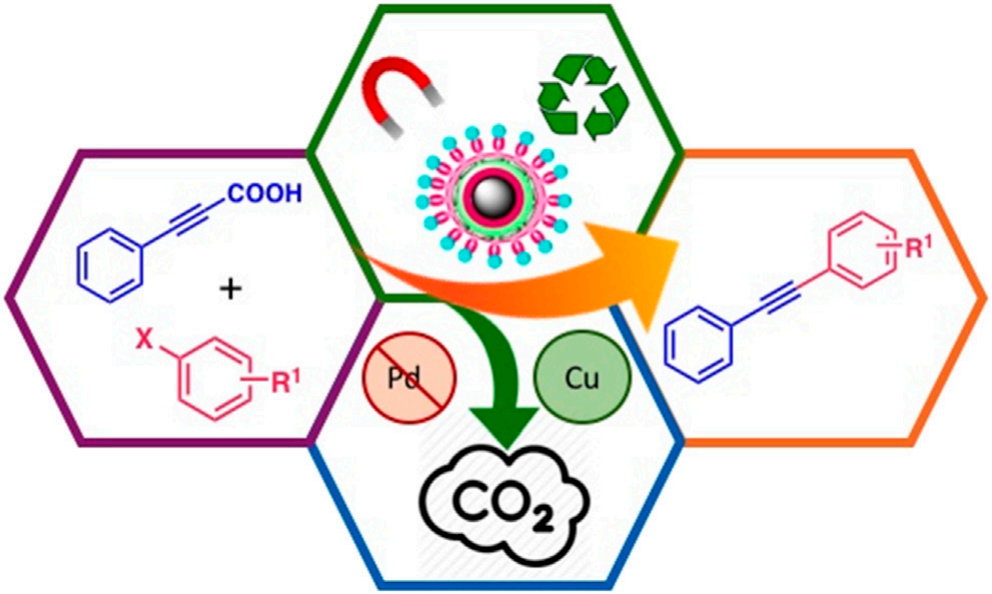
Magnetic Silica core-shell copper nanocatalyst in the decarboxylative coupling for the synthesis of alkynes.

## 1 Introduction

Transition metal catalyzed cross-coupling reactions for the construction of carbon-carbon (C-C) bond are amongst the most powerful and efficient strategy for synthesizing essential organic compounds including bioactive compounds, natural products, and polymeric materials (Sonogashira, 2002; Tykwinski, 2003; Jutand, 2004). For this, various traditional cross-coupling reactions have been employed, however, these methods utilize organometallic compounds that bear Mg, Al, Zn, Sn, B, and Si, which create problem of metal contamination in the product. On comparison with the well-established cross-coupling reactions, decarboxylative cross-coupling reaction offers several benefits since it involves readily available carboxylic acid derivatives that do not encounter storage and handling difficulties and releases less-toxic carbon dioxide as the by-product that reduces the waste treatment costs. (Moon et al., 2008a; Moon et al., 2008b; Kim and Lee, 2009; Park et al., 2010; Zhang et al., 2010; Zhao et al., 2010; Li et al., 2011; Pan et al., 2011; Qu et al., 2011; Li et al., 2012; Tartaggia et al., 2012; Park and Lee, 2013; Reddy et al., 2013; Lee et al., 2016; Maaliki et al., 2016).

Due to the wide occurrence of alkyne moiety in natural products, pharmaceuticals, and molecular materials, enormous efforts have been devoted towards the synthesis of arylalkynes and conjugated enynes.(Brandsma, 2003; Negishi and Anastasia, 2003; Stang and Tykwinski, 2006; Chinchilla and Nájera, 2007). Of all, the Sonogashira coupling superseded all the traditional methods for synthesizing internal alkynes from nucleophilic terminal acetylenes. (Nicolaou et al., 2005; Stang, 2008). However, the formation of homo-coupled by-product and volatile liquid nature of terminal alkynes, are the major drawbacks which limit their utility in industrial applications. (Kolarovic et al., 2011). Therefore, the straightforward synthesis of arylalkynes with some other readily available substrates remained a practical challenge for many years. In recent times, decarboxylative cross-coupling of alkynoic acids with haloarenes emerged as an attractive and practical solution. (Siemsen et al., 2000; Das et al., 2005; Feng and Loh, 2010; Jia and Jiao, 2010; Park et al., 2011).

To date, a number of homogeneous catalytic systems using palladium, copper and nickel catalysts have been developed for the decarboxylative coupling of alkynoic acids with haloarenes. (Edwin Raja et al., 2016). However, most of them employ toxic phosphine ligands, and costly additives that are also air and moisture sensitive.

Despite tremendous success in the development of this methodology, till now, only few Pd-based heterogeneous catalysts have been reported for this reaction. (Pyo et al., 2013; Reddy et al., 2016). Moreover, these protocols have common problems associated with the palladium based catalysts, such as their high cost that limit their industrial applications. Nonetheless, exploring cost-effective methods to prepare highly stable, efficient, and recyclable heterogeneous catalysts still remain a challenging task in this field.

Therefore, employment of an economic and greener first row transition metal heterogenized catalyst is highly desirable. In this respect, copper-catalyzed systems have gained tremendous progress due to their economic attractiveness and good functional group tolerance (Ley and Thomas, 2003; Evano et al., 2008; Monnier and Taillefer, 2009). However, copper mediated synthesis of internal alkynes *via* decarboxylative coupling reaction is still less explored (Shang et al., 2009).

In line with current challenges arising from the demands of industrial and fine chemistry, an ideal catalyst should not only possess high activity and selectivity towards the targeted products but should be stable, environmental friendly, recyclable, and must be easy to recover from the reaction mixture. In view of these requirements, silica coated magnetic nanoparticles (SMNPs) appear to be an ideal solution as solid supports due to their chemical inertness, robustness, easy magnetic recovery, recyclability, and environmentally benign nature (Rossi et al., 2014; Wang and Astruc, 2014; Sharma et al., 2016b).

Thus, in continuation of our ongoing research work on the development of nanocatalysts, and their applications in various organic transformations, (Sharma et al., 2015a; Sharma et al., 2015b; Sharma et al., 2016a; Sharma et al., 2016c; Arora et al., 2017; Gupta et al., 2017; Sharma et al., 2018), we herein describe the fabrication of a novel copper nanocatalyst with modified silica magnetic core-shell support for efficiently catalyzing decarboxylative coupling of alkynoic acid with haloarenes.

## 2 Materials and Methods

3-aminopropyltriethoxysilane (APTES), tetraethoxyorthosilicate (TEOS), and 4, 5-diazafluoren-9-one were procured from Sigma Aldrich. Ferric sulphate hydrate and ferrous sulphate heptahydrate were obtained from Sisco Research Laboratory (SRL). copper(I) iodide, Cs_2_CO_3_, and toluene were purchased from Merck.

The prepared nanocatalyst was characterized using several techniques. X-ray diffraction (XRD) patterns were obtained from a D8 Discover Bruker AXS (Karlsruhe, Bundesland, Germany) diffractometer in the 2θ range of 10–80. For uniformity and morphology HR-TEM, FEI TECNAIF 30 transmission electron microscope with HAADF detector was used and operated at 300 kV. In order to study the chemical composition of the catalyst, X-ray energy dispersive spectroscopy (EDS) was carried out using Ametek EDAX system. Carl Zeiss India scanning electron microscope was used to investigate for analyzing the structural properties of prepared nanocomposites. EV-9, Microsense, ADE vibrating sample magnetometer was used to conduct magnetization measurements. The Fourier transform infrared spectra (FT-IR) of NPs were collected at every stage of synthesis using Perkin-Elmer Spectrum 2000. For the estimation of amount of copper in the catalyst and in the supernatant inductively coupled plasma (ICP) of PerkinElmer Optima 2100 DV was used. The products were confirmed by making use of Agilent gas chromatography-mass spectrometer with a HP-5MS 5% phenyl methyl siloxane capillary column (30.0 m × 0.25 mm × 0.25 μm) using helium as a carrier gas.

### 2.1 Synthesis of Cu-DF@ASMNPs

**SCHEME 1:**
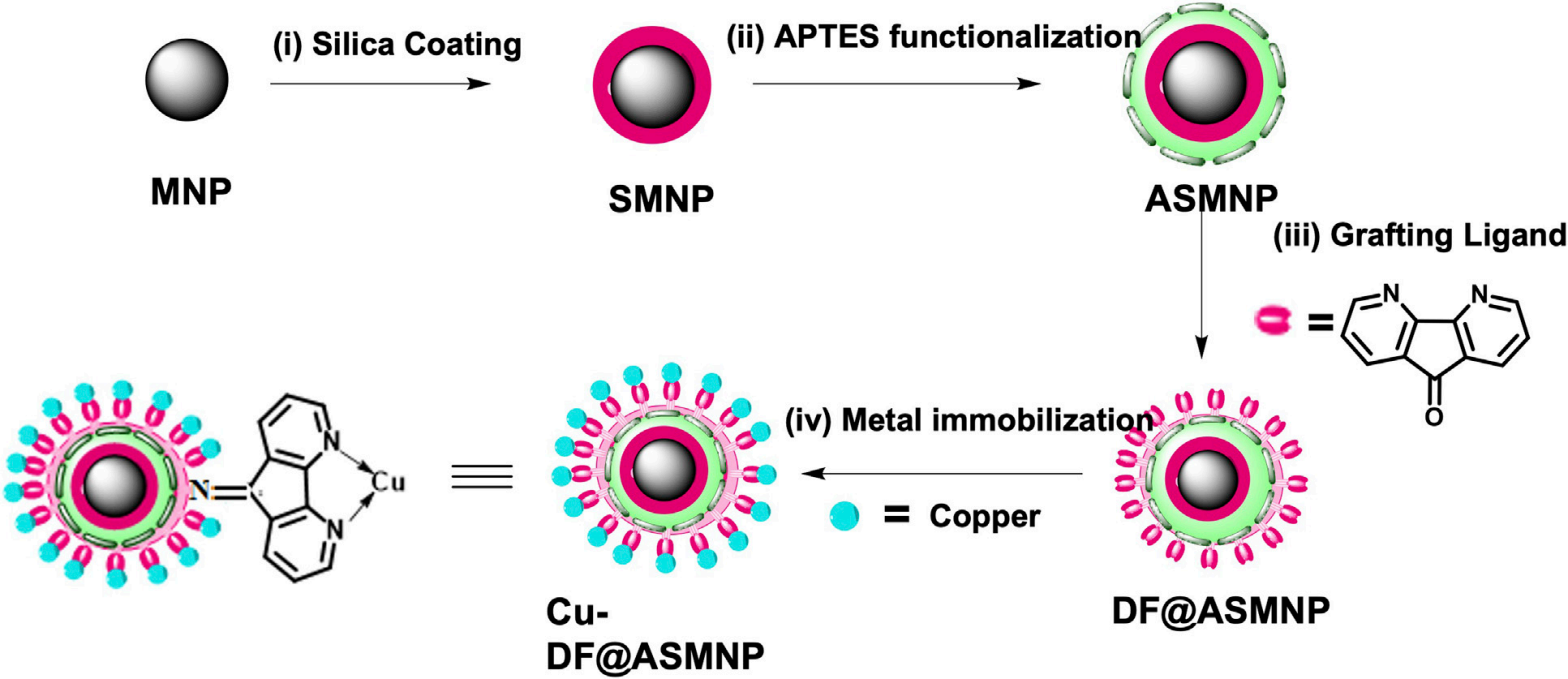
Scheme for the fabricating Cu-DF@ASMNPs core-shell nano-catalyst.

Firstly, MNPs were synthesized by co-precipitation technique. (Polshettiwar and Varma, 2009). For this, ferric sulphate (6.0 g) and ferrous sulphate (4.2 g) were dissolved in 250 ml distilled water and stirred at 60 C. To the obtained orange solution, 25% of NH_4_OH (15 ml) was added dropwise and the solution was stirred vigorously for 30 min. The obtained black precipitates of MNPs were separated with external magnet and thoroughly washed with water and ethanol and finally dried under vacuum. On to this, silica coating was performed *via* sol-gel approach to form SMNPs, (Zhang et al., 2011), which was further functionalized with the NH_2_ linker, APTES. For silica coating, 0.5 g of MNPs were dissolved in 2.2 ml of 0.1 M HCl and dispersed in 200 ml ethanol and 50 ml water under sonication. Further, 5 ml NH_4_OH was added followed by addition of 1 ml of TEOS under constant stirring at 60 C for 6 h to give SMNPs. These SMNPs were washed with ethanol and water. The functionalization with NH_2_ linker was performed by adding 0.5 ml of APTES to the dispersed solution of 0.1 g of SMNPs in 100 ml of ethanol under constant stirring at 50 C for 6 h. 1 g of resulting APTES functionalized SMNPs (ASMNPs) were further reacted with a 0.75 mmol of bidentate ligand, 4, 5-diazafluoren-9-one (DF) in acetone at 70°C for 3 h. The resulting DF@ASMNPs were washed with ethanol and dried under vacuum. To 1 g of DF@ASMNPs, 1.5 mmol of copper iodide was added and the solution was stirred for 3 h using acetone as solvent. The resulting nanocatalyst was magnetically recovered and thoroughly washed with deionized water and dried under vacuum to obtain the final catalyst Cu-DF@ASMNPs. (Scheme 1).

### 2.2 Cu-DF@ASMNPs Catalyzed Internal Alkynes Synthesis

For this, 10 ml of round bottom flask was flushed with nitrogen and to this, haloarene (0.5 mmol), alkynoic acid (0.6 mmol), Cu-DF@ASMNPs (25 mg), and Cs_2_CO_3_ (1.0 mmol) were added. Again, nitrogen was flushed, and toluene (2 ml) was added at room temperature. The temperature was raised to 100°C with continuous stirring for 12 h. On bringing to room temperature, the mixture was extracted with ethyl acetate. The reaction was monitored and analyzed by GC-MS.

## 3 Results and Discussion

### 3.1 Characterization of Catalyst

#### 3.1.1 FT-IR Spectroscopy

**FIGURE 1:**
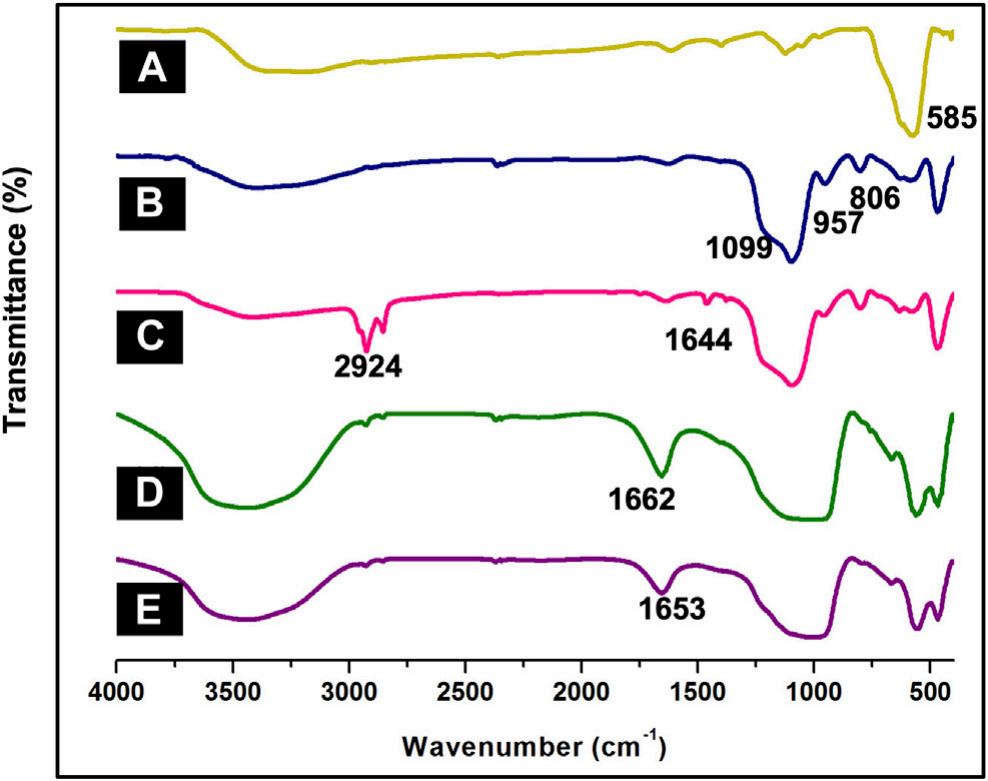
FT-IR spectra of **(A)** MNPs, **(B)** SMNPs, **(C)** ASMNPs, **(D)** DF@ASMNPs, and **(E)** Cu-DF@ASMNPs.

In order to analyze parent nanocomposite and its further functionalization, FT-IR spectroscopy was employed. A band was observed at 585 cm^−1^ in the IR-spectrum of MNPs depicting the Fe-O stretching absorption (Figure 1A). (Zhu et al., 2011) The intensity of this band reduced on silica-coating with the appearance of three new sharp bands in the region of 806, 957 and 1,099 cm^−1^, corresponding to the symmetric Si-O-Si, symmetric Si-O(H) and asymmetric Si-O-Si stretching vibrations respectively (Figure 1B) (Kooti and Afshari, 2012) Further functionalization of SMNPs with APTES resulted in absorption at 2,924 cm^−1^ and 1,644 cm^−1^, which corresponds to CH_2_ and NH_2_ from aminopropyl moiety of APTES (Figure 1C) (Yamaura et al., 2004) The immobilization of ligand DF onto ASMNPs was confirmed by the band at 1,662 cm^−1^ accredited to C=N stretching frequency (Figure 1D) and to this, metal was immobilized using CuI which shifted the prominent band at 1,662 cm^−1^ to a lower wavenumber indicating strong metal-ligand interaction (Figure 1E). (Masteri-Farahani and Tayyebi, 2011; Esmaeilpour et al., 2012).

#### 3.1.2 XRD Studies

**FIGURE 2:**
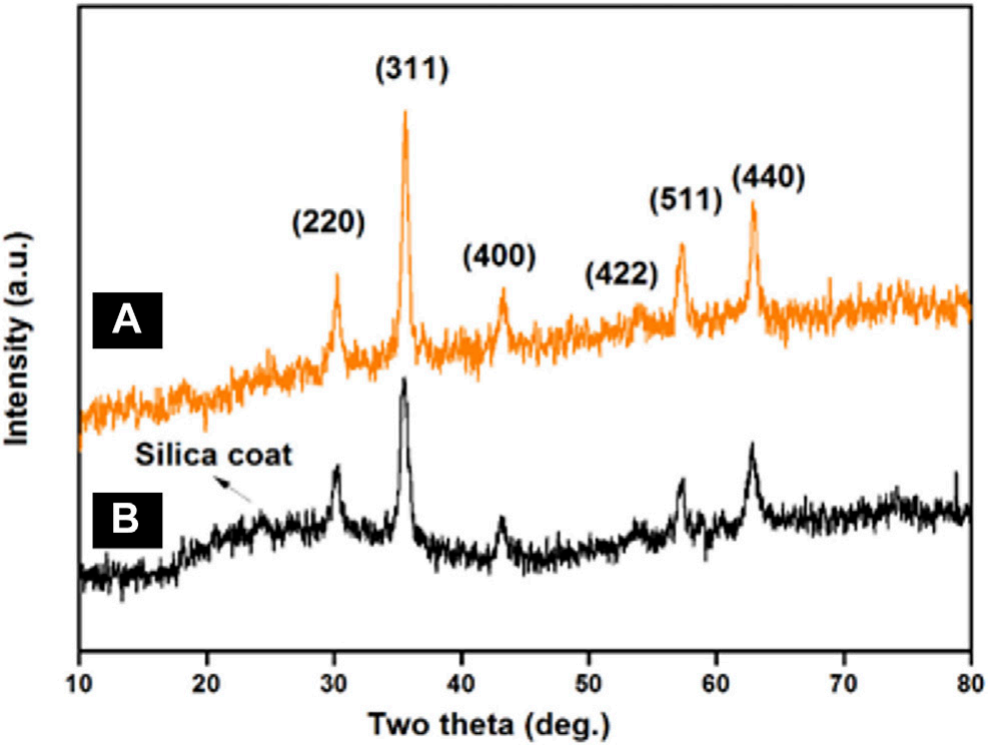
XRD patterns of **(A)** MNPs and **(B)** SMNPs.

To assess the crystalline nature of synthesized MNPs and SMNPs, powder X-Ray diffraction measurements were carried out. For MNPs (Figure 2A), six characteristic peaks were observed at 2θ: 30.366^o^, 35.663^o^, 43.024^o^, 53.6^o^, 57.299^o^, and 62.865^o^ corresponding to the (220), (311), (400), (422), (511) and (440) crystallographic faces of magnetite (Abu-Reziq and Alper, 2012). These peaks were in accordance with the standard XRD data provided by the Joint Committee on Powder Diffraction Standards (JCPDS) card number 19–0,629 and is ascribed to inverse cubic spinel Fe_3_O_4_ crystal (Abu-Reziq et al., 2006). The average crystallite size of the MNPs was calculated by the Scherrer equation {D_hkl_ = Kλ/(/(β_hkl_cosθ)}, where D_hkl_ represents the size of the axis parallel to the (hkl) plane, k is a constant with a common value of 0.89 for spherical particles, *λ* is the wavelength of radiation, *β*_hkl_ is the full-width at half-maximum (FWHM) in radians, and θ is the diffraction angle. The mean crystallite size was found to be ∼10.6 nm for the (311) reflection. Besides these six diffraction peaks, a weak broad hump at 2θ = 20–24^o^ is observed in the XRD pattern of SMNPs showed in Figure 2B, which is attributed to amorphous silica (Zhang et al., 2012).

#### 3.1.3 SEM Analysis

**FIGURE 3:**
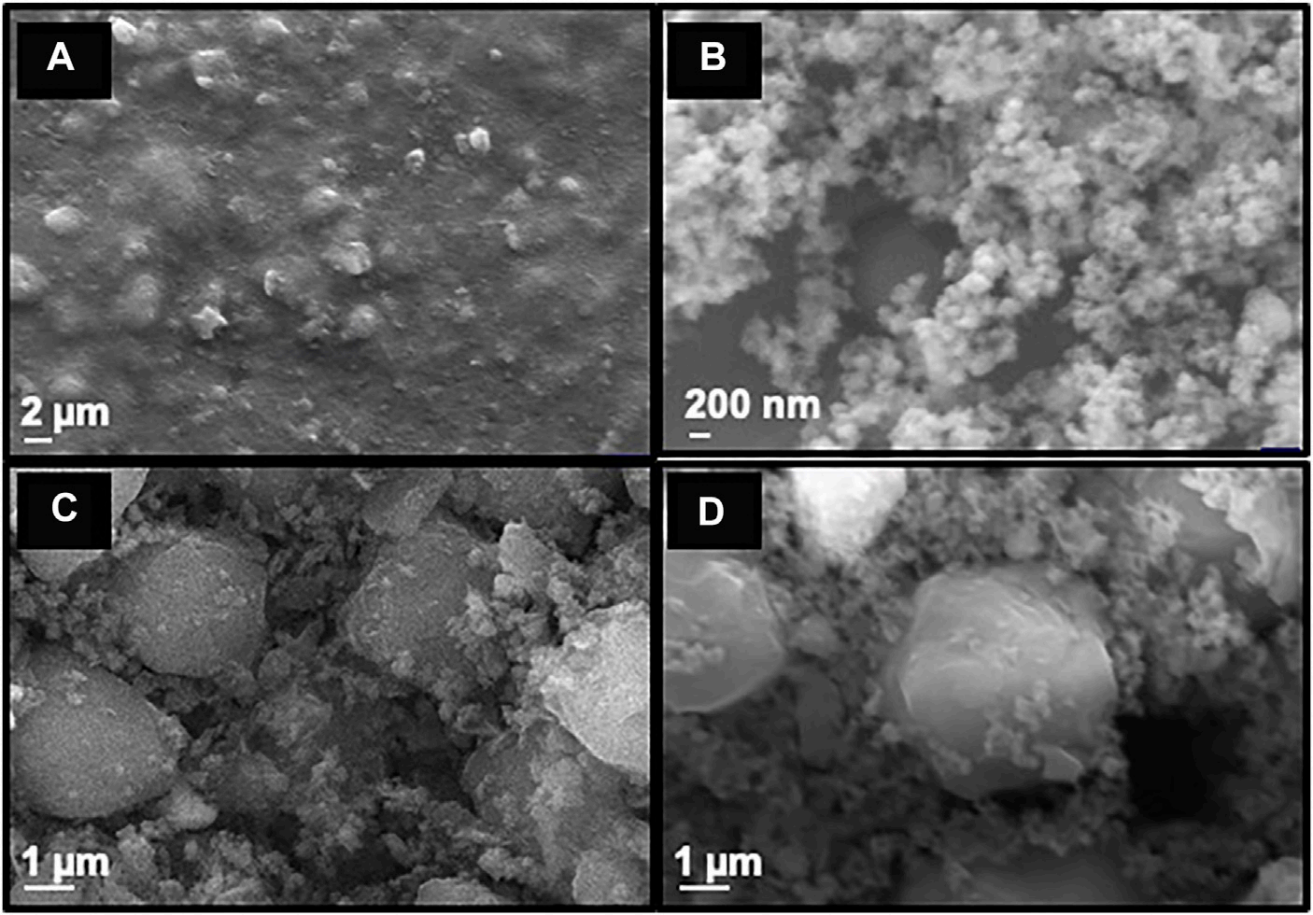
SEM images of **(A)** MNPs, **(B)** SMNPs, **(C)** Fresh Cu-DF@ASMNPs and **(D)** Recovered Cu-DF@ASMNPs.

To investigate the topography of the synthesized nanoparticles, scanning electron microscopic (SEM) analysis was performed and it was found that the smooth surface of MNP (Figure 3A) turns spongy on silica coating (Figure 3B). The spherical morphology of the final Cu-DF@ASMNPs catalyst was seen with slight agglomeration and appears the same as that of SMNP (Figure 3C). This suggested that the surface modification methods did not alter the morphology of the nanocatalyst. Besides this, the SEM image of the recovered catalyst (Figure 3D) also indicates that the reaction did not affect the morphology of the catalyst.

#### 3.1.4 TEM Analysis

**FIGURE 4:**
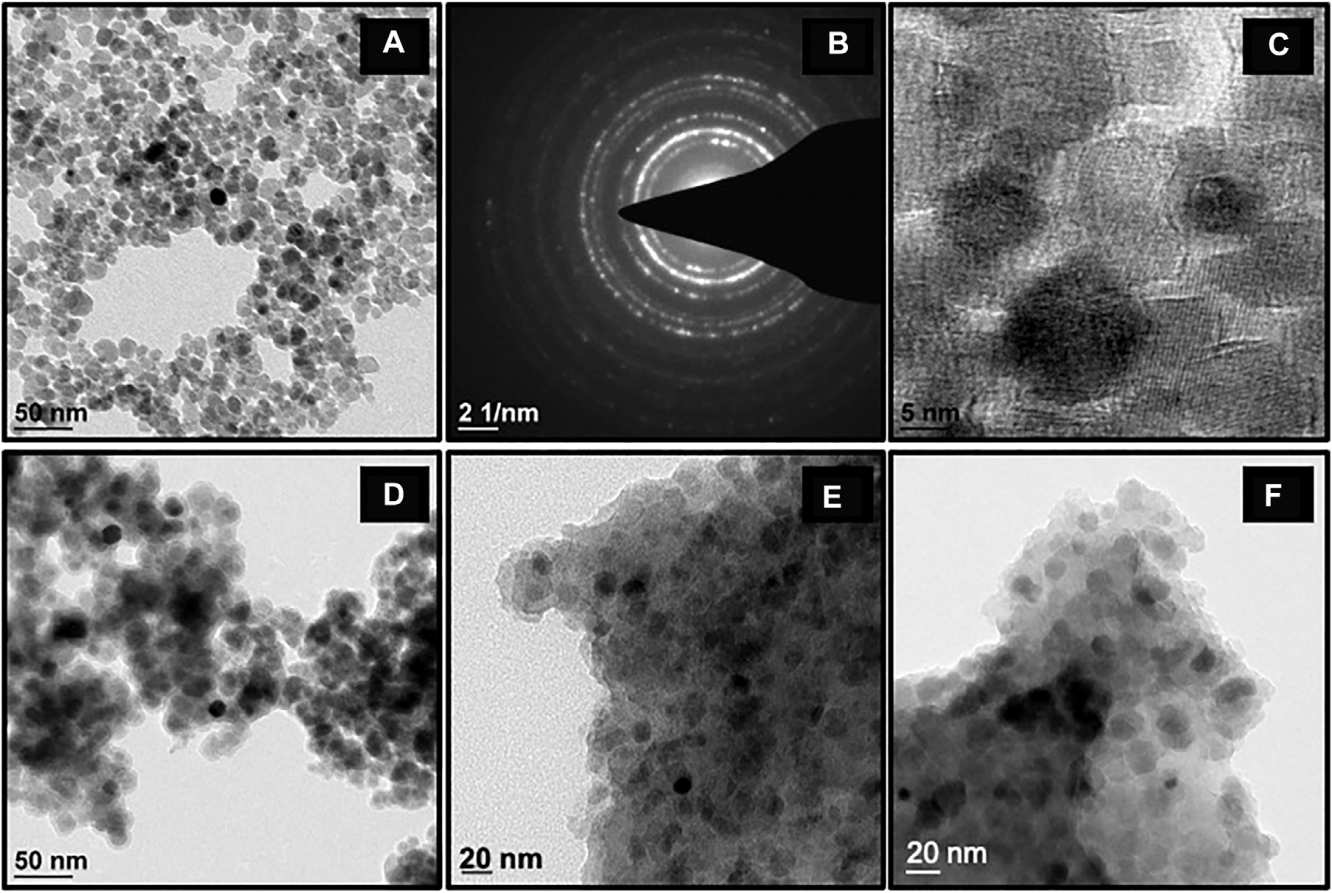
TEM images of the nanoparticles obtained at different stages of synthesis: **(A)** MNPs, **(B)** SAED pattern of MNPs, **(C)** HR-TEM image of MNPs, **(D)** SMNPs, **(E)** Fresh Cu-DF@ASMNPs, and **(F)** Recovered Cu-DF@ASMNPs.

TEM studies were performed to study the morphological changes of the synthesized nanomaterials, Figure 4A depicts that MNPs are polydisperse in nature and display slight agglomeration (Wang et al., 2010; Wang et al., 2013). An array of bright diffraction rings was observed in the selected area electron diffraction pattern (SAED) (Figure 4B) which confirmed the crystalline nature of these nanocomposites and also in accordance with the resultant XRD pattern. The average interplanar distance of the MNPs was measured from a high-resolution transmission electron microscopy (HR-TEM) image and was found to be ∼0.20 nm, which correlates with the (311) plane of inverse spinel Fe_3_O_4_ structure (Figure 4C). A dark core-shell of MNP, with an almost uniform silica coat of 4–5 nm thickness, was observed in the TEM image of SMNP (Figure 4D). TEM images of final catalyst and recovered catalyst are shown in Figure 4E and Figure 4F respectively, which further confirm that the structural morphology remain unchanged after the coupling reaction. In order to find the average particle size of MNPs, 52 colloidal aggregates were analyzed and it was found to be in the range of 10–11 nm (Supplementary Figure S1) which is in well accordance with the XRD results.

#### 3.1.5 EDS Analysis and Metal Content Determination

**FIGURE 5:**
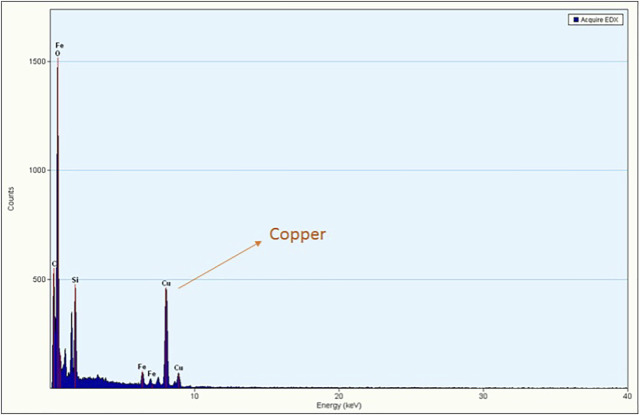
EDS pattern of Cu-DF@ASMNPs.

Energy dispersive X-ray analysis was performed to detect the composition of the synthesized nanocomposites, and the EDS spectrum displayed well-defined peaks of copper, silicon and iron (Figure 5) that substantiate the effective grafting of copper on the Cu-DF@ASMNPs. Moreover, to determine the amount of copper present in the final catalyst, ICP analysis was conducted and the metal loading was found to be 0.3217 mmolg^−1^.

#### 3.1.6 VSM Analysis

**FIGURE 6:**
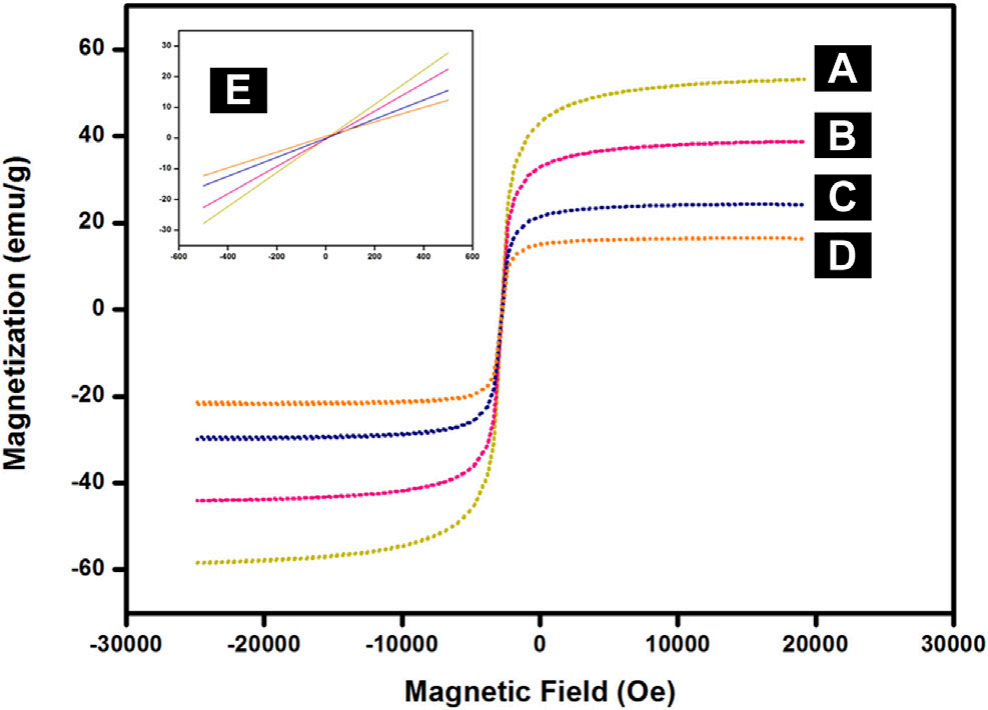
Magnetization curves for **(A)** MNPs, **(B)** SMNPs, **(C)** ASMNPs, **(D)** Cu-DF@ASMNPs and **(E)** inset: enlarged image near the coercive field.

The field-dependent magnetization measurement of synthesized nanocomposites is provided in Figure 6. The superparamagnetic behaviour of these nanoparticles was confirmed by their magnetization curves which display no hysteresis at room temperature. This was further corroborated by the inset in Figure 6 where both coercivity and remanence were negligible in the absence of an externally applied magnetic field. The saturation magnetization value of MNPs, SMNPs, ASMNPs, and Cu-DF@ASMNPs were found to be 59 emu g^−1^, 40 emu g^−1^, 29 emu g^−1^, and 19 emu g^−1^ respectively. This decrease in the M_s_ value is due to the non-magnetic nature of the functionalizing groups. (Hu et al., 2005; Digigow et al., 2014). Despite of lower value of magnetization, the net magnetism of Cu-DF@ASMNPs was high enough for its effortless removal *via* an external magnet.

### 3.2 Catalytic Studies

#### 3.2.1 Optimization of the Reaction Conditions

**FIGURE 7:**
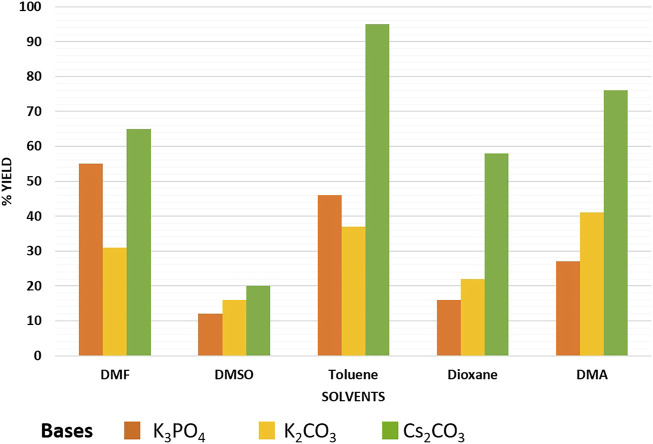
Effect of base and solvent on synthesis of internal alkynes [Reaction conditions: iodobenzene (0.5 mmol), phenylpropiolic acid (0.6 mmol), Cu-DF@ASMNPs (25 mg), base (1.0 mmol), solvent (2 ml), 100°C, 12 h, under N_2_].

In order to test the efficacy of the prepared nano-catalyst, Cu-DF@ASMNPs and to discover the optimal reaction conditions, phenylpropiolic acid (0.6 mmol) and iodobenzene (0.5 mmol) were selected as the coupling partners. For the optimization of decarboxylative cross-coupling reaction, reaction parameters like solvent, base and catalytic amount were assessed. Figure 7 represent several combinations of base and solvent, and out of them highest yield was obtained when the base was Cs_2_CO_3_ (1 mmol), and toluene (2 ml) was the solvent. For the determination of the optimal catalytic amount, a blank test was carried out, where no significant yield was obtained. Although, the reaction gave product with 10 mg of catalyst and on increasing the amount of catalyst, significant increase in yield of the product was noticed. However, no noticeable increase in the product yield was found when 30 mg of catalyst was used and the best yield was achieved with 25 mg of catalyst (Supplementary Table S1). Also, the reaction was performed under diverse range of temperatures while keeping other parameters constant and 100 C was found to be the optimum temperature to carry out the coupling with 25 mg of synthesized catalyst (Supplementary Figure S2). Therefore, all the reactions were performed using toluene as the reaction solvent, Cs_2_CO_3_ as the base for 12 h at 100 C in the presence of 25 mg of Cu-DF@ASMNPs under N_2_ atmosphere.

#### 3.2.2 Catalytic Activity of Cu-DF@ASMNPs Catalyst for Decarboxylative Cross-Coupling of Alkynoic Acids With Haloarenes

To demonstrate the efficiency of this method, various haloarenes including iodo and bromo derivatives were coupled with phenylpropiolic acid using the optimized reaction conditions (Table 1). To check the scope of this reaction, we initially examined a variety of iodoarenes possessing both activating and deactivating groups including methoxy, methyl, naphthyl, nitro, and chloro. It was observed that the reaction went smoothly for both electron donating and withdrawing groups on the iodoarene and excellent yields were obtained for various internal alkynes. For *p*-iodotoluene and *p*-iodoanisole the yield was similar, 90 and 88% respectively (entries 2 and 3). However, slight increase in yield was obtained when haloarene bearing electron withdrawing substituent was employed (entry 5).

**TABLE 1:** Scope of catalytic performance of the Cu-DF@ASMNP for synthesizing internal alkynes^a^. 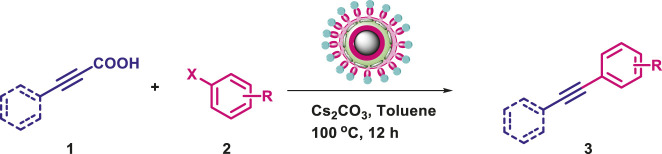

Entry	Haloarenes	Product	Yield^b^ (%)	TON^c^
1	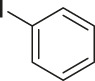	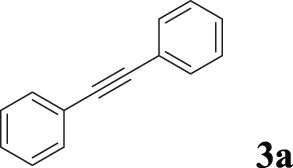 **3a**	92	115
2	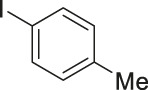	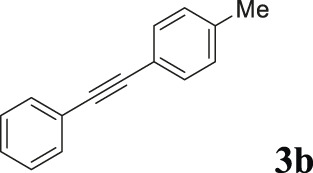 **3b**	90	113
3^d^	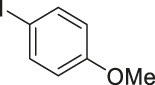	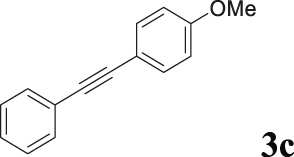 **3c**	88/82^e^	110
4	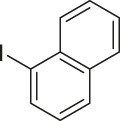	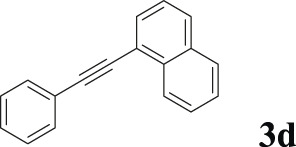 **3d**	94/90^e^	118
5	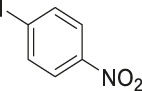	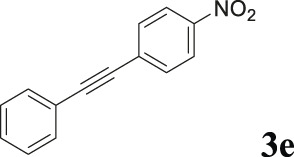 **3e**	95	119
6	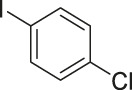	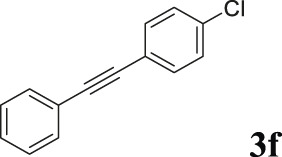 **3f**	89/85^e^	111
7	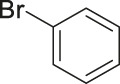	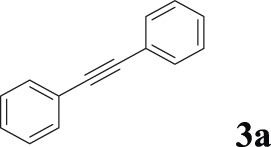 **3a**	84	105
8	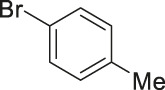	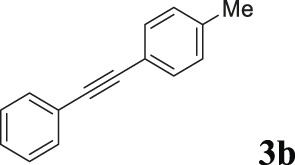 **3b**	86	108
9	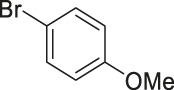	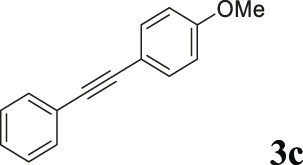 **3c**	80	100
10	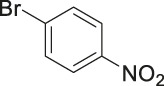	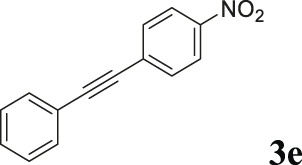 **3e**	88/80^e^	110
11	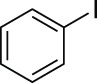	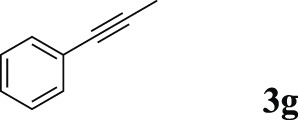 **3g**	68	85

a Reaction conditions: Haloarene (0.5 mmol), alkynoic acid (0.6 mmol), Cu-DF@ASMNP (25 mg), Cs_2_CO_3_ (1.0 mmol), toluene (2 ml), 100°C, 12 h, under N_2_.

b GC-MS, yield.

c TON , Calculated using the 0.3217 mmolg^−1,^ copper.

e Isolated yield.

d Reaction was performed on large scale; Haloarene (5 mmol), alkynoic acid (6 mmol), Cu-DF@ASMNP (0.25 g), Cs_2_CO_3_ (10.0 mmol), toluene (10 ml), 100°C, 12 h, under N_2_.

To gauge the efficacy of reaction, more practical coupling partner aryl bromides were used. To our delight they also worked very well for this coupling reaction and both electron withdrawing as well as electron donating aryl bromides were efficiently converted into corresponding internal alkynes in excellent yields. To further assess the potential of the catalyst, an aliphatic alkynoic acid, 2-butynoic acid (entry 11) was tested for this reaction but this afforded slightly lower yield. Isolated yields were obtained for few selected reactions consisting of haloarene bearing electron donating group (entry 3), neutral group (entry 4), a halogen substituent (entry 6) to check for any kind of interference, and electron withdrawing group (entry 10).

#### 3.2.3 Catalytic Stability and Reusability

**FIGURE 8:**
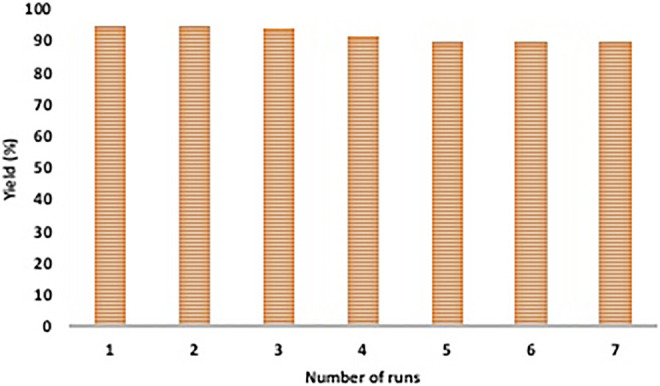
Catalyst recycling test for the synthesis of internal alkynes.

To test the reusability of catalyst, after each experiment (conducted under optimized conditions), the catalyst was magnetically separated, washed with ethyl acetate and ethanol and dried under vacuum. This was then used to perform subsequent reactions. It was observed that the catalyst was active up to seven runs without any notable decrease in its performance (Figure 8). SEM and TEM images further confirmed the unaltered structure and morphology of the recovered catalyst (Figure 3D and Figure 4F).

#### 3.2.4 Heterogeneity Test

In order to estimate the leaching rate and heterogeneous nature of the catalyst, two sets of corresponding experiments were conducted. A standard reaction was conducted for the first set where the catalyst was magnetically removed after completion of reaction, and filtrate was analyzed under ICP, which showed no leaching. A split test was performed with the second set, in which the standard reaction was conducted with catalyst for 2 hours, which roughly corresponds to 20% conversion by GC-MS. Afterwards, the nanocatalyst was magentically separated from the reaction mixture and the reaction was further pursued. No coupling product in the reaction mixture was observed up to 10 hours under the same reaction conditions, which authenticate the truly heterogeneous nature of the nanocatalyst.

#### 3.2.5 Plausible Mechanism

**FIGURE 9:**
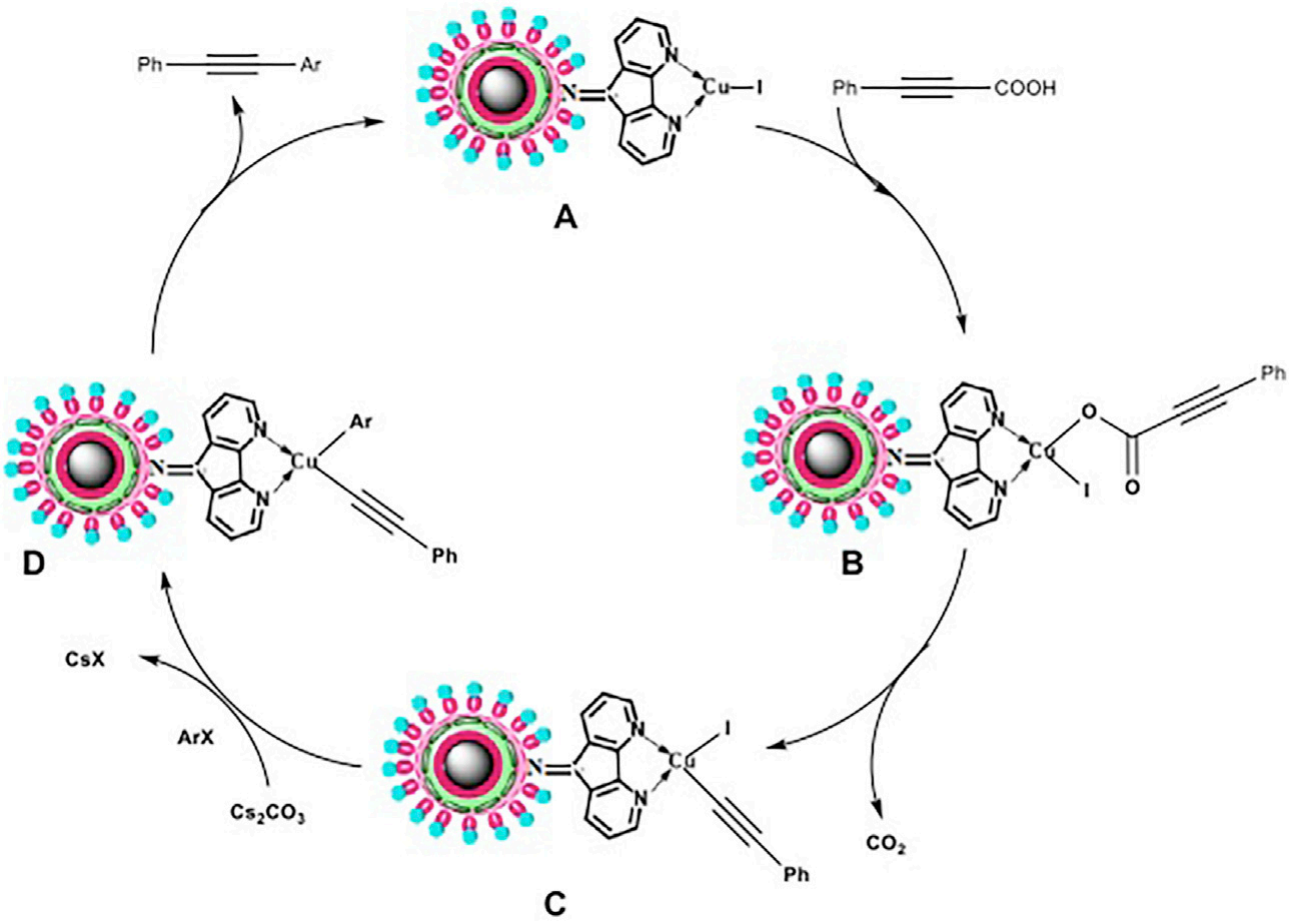
Proposed reaction mechanism.

Figure 9 depicts the proposed mechanism that has been derived from earlier reports. (Okuro et al., 1993; Ray et al., 2008; Gonda et al., 2010; Lauterbach et al., 2010). The reaction between Cu-DF@ASMNPs A and alkynoic acid produces intermediate B, which undergoes decarboxylation to yield C, an alkynyl copper intermediate. Further addition of haloarene results in the formation of another intermediate D, which then undergoes reductive elimination, to give the product while regenerating the catalyst A.

Finally, in order to show the superiority of the synthesized catalyst, we compared our obtained results with the previously reported work (Table 2) and found that our catalyst was far more efficient in terms of reaction conditions, reaction time and catalytic recovery. Also, it is the first copper based heterogeneous system for synthesis of internal alkynes.

**TABLE 2:** A comparison of the obtained results with previous published work for the synthesis of internal alkynes.

S.No	Acid	Coupling partner	Catalyst	Conditions	Yield (%)	Ref
1	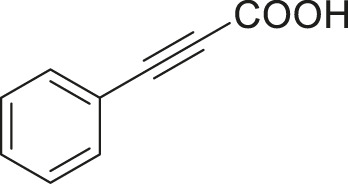	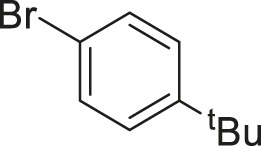	Pd_2_dba_3_ (5 mol%)	dppf (10 mol%), TBAF (6.0 equiv), NMP, 90°C, 1 h	88	Moon et al. (2008b)
2	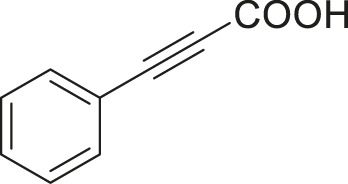	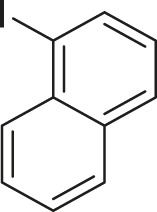	Pd_2_dba_3_ (2 mol%)	PPh_3_ (16 mol%), Ag_2_O (1–3 equiv), LiI (3–6 equiv), DMF	64	Kim and Lee, (2009)
3	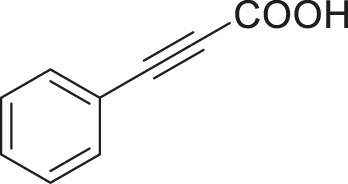	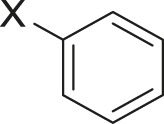	Pd (OAc)_2_	XPhos, Cs_2_CO_3_, THF, 80°C	70–95	Zhang et al. (2010)
4	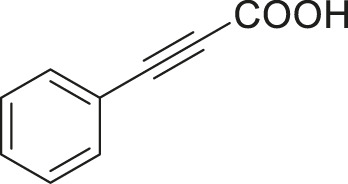	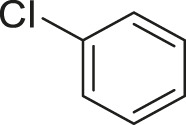	Palladacycle (1 mol%)	Xphos (4 mol%), K_2_CO_3_ (2 equiv), xylene/H_2_O 120°C, 3 h	94	Li et al. (2013)
5	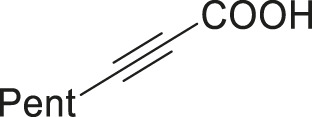	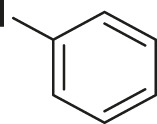	Pd (PPh_3_)_2_Cl_2_ (1 mol%)	2 mol% of dppb, DMSO, 110°C, 2 h	96	Moon et al. (2008a)
6	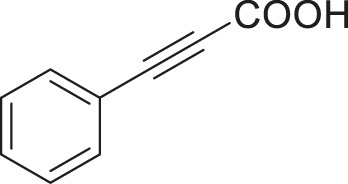	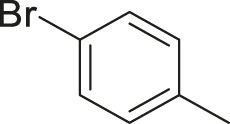	[PdCl (allyl)]_2_ (2.5 mol%)	SPhos (7.5 mol%), TBAF (3.0 equiv), NMP/H_2_O, 80°C, 14 h	84	Tartaggia et al. (2012)
7	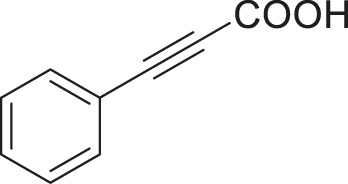	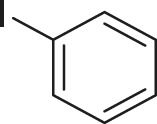	CuI (10 mol%)	1,10-Phen (10 mol%), Cs_2_CO_3_ (1.5 equiv), DMF, 130°C, 24 h	99	Zhao et al. (2010)
8	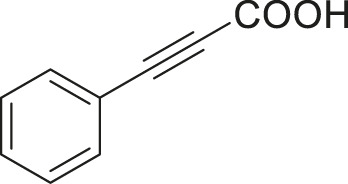	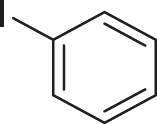	CuI (2 mol%)	PPh_3_ (4 mol%), K_2_CO_3_ (3 equiv), DMSO/H_2_O 100°C, 24 h, under argon	97	Li et al. (2012)
9	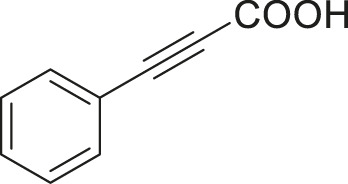	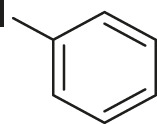	CuI (0.5 mol%)/Fe (acac)_3_) (1 equiv)	K_3_PO_4_ (2 equiv), DMSO (2 ml), 140°C, 24–48 h, under argon	98	Li et al. (2011)
10	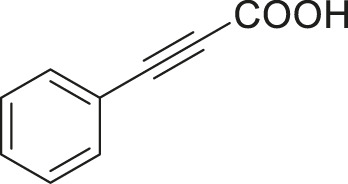	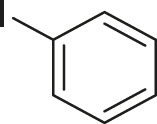	CuSO_4_·5H_2_O (10 mol%)	L (10 mol%), K_2_CO_3_(2 equiv), DMF, 130°C	90	Wang et al. (2016)
11	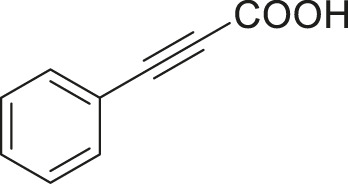	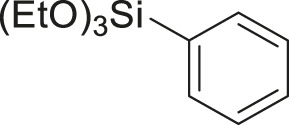	Ni (acac)_2_ (10 mol%)	1,10-Phen (10 mol%), CsF(1 equiv), CuF_2_(1 Equiv)	90	Edwin Raja et al. (2016)
12^a^	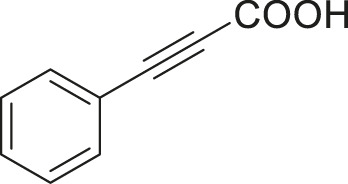	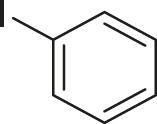	Pd-CNT (5 mol%)	DBU (2 equiv.), DMSO, 90°C, 12 h	95	Pyo et al. (2013)
13^a^	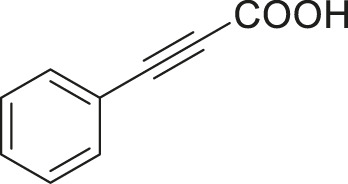	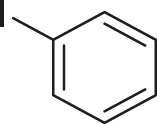	Pd@PS (3 mol%)	DBU (3 equiv), DMF, 110°C, 12 h	66	Reddy et al. (2016)
14^a^	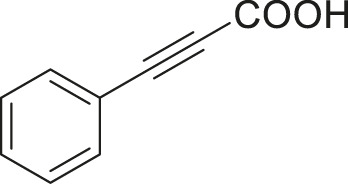	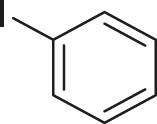	Cu-DF@ ASMNP	Cs_2_CO_3_, toluene, 100°C, 12 h	92	This work

a Heterogeneous catalyst.

In summary, a highly effective palladium-free Cu-DF@ASMNPs nanocatalyst was fabricated successfully and applied towards the synthesis of internal alkynes *via* decarboxylative cross-coupling reaction. These nanocomposites endowed low metal loading, high stability, and good functional group tolerance with excellent yields and high turn-over numbers. It is noteworthy that this catalytic system is the first report of copper based magnetic nanocatalyst that represents a practical and low-cost route to prepare internal alkynes. In addition, the effortless magnetic recovery and reusability of the catalyst for at least seven runs without any marked loss in its performance makes it an efficient protocol to produce a wide variety of unsymmetrical alkynes.

## Data Availability

The original contributions presented in the study are included in the article/Supplementary Material, further inquiries can be directed to the corresponding author.
