# Supplementary material for: An Efficient and Sustainable Approach to Decarboxylative Cross-Coupling Using Silica Coated Magnetic Copper Nanocatalyst for the Synthesis of Internal Alkynes
Source: Front Chem. 2022 Jan 17;9:773855. doi: 10.3389/fchem.2021.773855 (PMC8802109; doi:10.3389/fchem.2021.773855)
Supplement: Supplementary file 1 [file DataSheet1.DOCX]

Supplementary Material


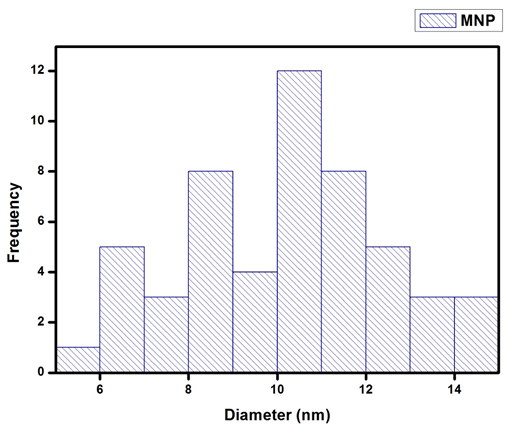


**Supplementary Figure 1.** Size distribution histogram for MNPs.

**Supplementary Table S1** Screening of copper catalysts for the synthesis of internal alkynes^a^

| **Entry** | **Catalyst** | **Amount  (mg)** | **Time  (h)** | **Yield   (%)^b^** |
| --- | --- | --- | --- | --- |
| 1. | No | - | 10 | Trace |
| 2. | CuI (5 mol%) | 5 | 10 | 49 |
| 3.^c^ | CuI (5 mol%) | 5 | 10 | 84 |
| 4.^c^ | CuI (5 mol%) | 5 | 12 | 90 |
| 5. | Cu-DF@ASMNPs | 10 | 12 | 61 |
| 6. | Cu-DF@ASMNPs | 20 | 12 | 83 |
| 7. | Cu-DF@ASMNPs | 25 | 12 | 92 |
| 8. | Cu-DF@ASMNPs | 30 | 12 | 92 |

^a^Iodobenzene (0.5 mmol), phenylpropiolic acid (0.6 mmol), Cs_2_CO_3_ (1.0 mmol), toluene (2 mL), 100 °C, under N_2_ atmosphere.

^b^Yield was determined by GC-MS.

^c^Reaction performed with 1,10-phenanthroline (5 mol%).


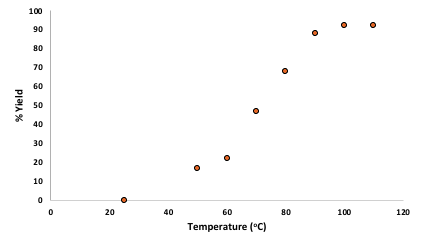


**Supplementary Figure 2.** Effect of temperature on model reaction of decarboxylative cross-coupling.

**General remarks**

^1^H and ^13^C NMR spectra were recorded on 400 MHz NMR and 100 MHz NMR spectrometers and CDCl_3_ (from Eurisotop) was used as a solvent. The chemical shifts are reported in ppm considering CDCl_3_ as an internal standard for ^1^H (*δ* 7.26 ppm) and ^13^C NMR (*δ* 77.00 ppm). Signal patterns are indicated as s, singlet; d, doublet; dd, doublet of doublets; t, triplet; m, multiplet; bs, broad singlet. Coupling constants (*J*) are given in hertz (Hz).

**1-methoxy-4-(phenylethynyl)benzene**

^1^H NMR (400 MHz, CDCl_3_): *δ* 3.85 (s, 3H), 6.93 (d, *J* = 8.8 Hz, 2H), 7.36-7.42 (m, 3H), 7.54 (d, *J* = 8.8 Hz, 2H), 7.59 (dd, *J* = 7.6 Hz, *J* = 1.6 Hz, 2H); ^13^C {^1^H} NMR (100 MHz, CDCl_3_): *δ* 55.3, 88.2, 89.5, 114.1, 115.4, 123.7, 128.0, 128.4, 131.5, 133.1, 159.7.

**1-chloro-4-(phenylethynyl)benzene**

^1^H NMR (400 MHz, CDCl_3_): *δ* 7.36 (d, *J* = 8.4 Hz, 2H), 7.38-7.40 (m, 3H), 7.49 (d, *J* = 8.4 Hz, 2H), 7.56-7.58 (m, 2H); ^13^C {^1^H} NMR (100 MHz, CDCl_3_): *δ* 88.3, 90.4, 121.8, 123.0, 128.4, 128.5, 128.7, 131.6, 132.8, 134.3.

**1-nitro-4-(phenylethynyl)benzene**

^1^H NMR (400 MHz, CDCl_3_): *δ* 7.41-7.42 (m, 3H), 7.57-7.60 (m, 2H), 7.66 (d, *J* = 8.8 Hz, 2H), 8.21 (d, *J* = 8.8 Hz, 2H); ^13^C {^1^H} NMR (100 MHz, CDCl_3_): *δ* 87.6, 94.7, 122.1, 123.6, 128.6, 129.3, 130.2, 131.9, 132.3, 147.0.

**1-(phenylethynyl)naphthalene**

^1^H NMR (400 MHz, CDCl_3_): *δ* 7.44-7.49 (m, 3H), 7.51-7.55 (m, 1H), 7.59-7.62 (m, 1H), 7.66-7.70 (m, 1H), 7.73-7.75 (m, 2H), 7.85 (d, *J* = 7.6 Hz, 2H), 7.90-7.94 (m, 2H), 8.55 (d, *J* = 7.6 Hz, 2H); ^13^C {^1^H} NMR (100 MHz, CDCl_3_): 87.6, 94.4, 121.0, 123.5, 125.3, 126.3, 126.5, 126.8, 130.4, 131.7, 133.3, 133.4.


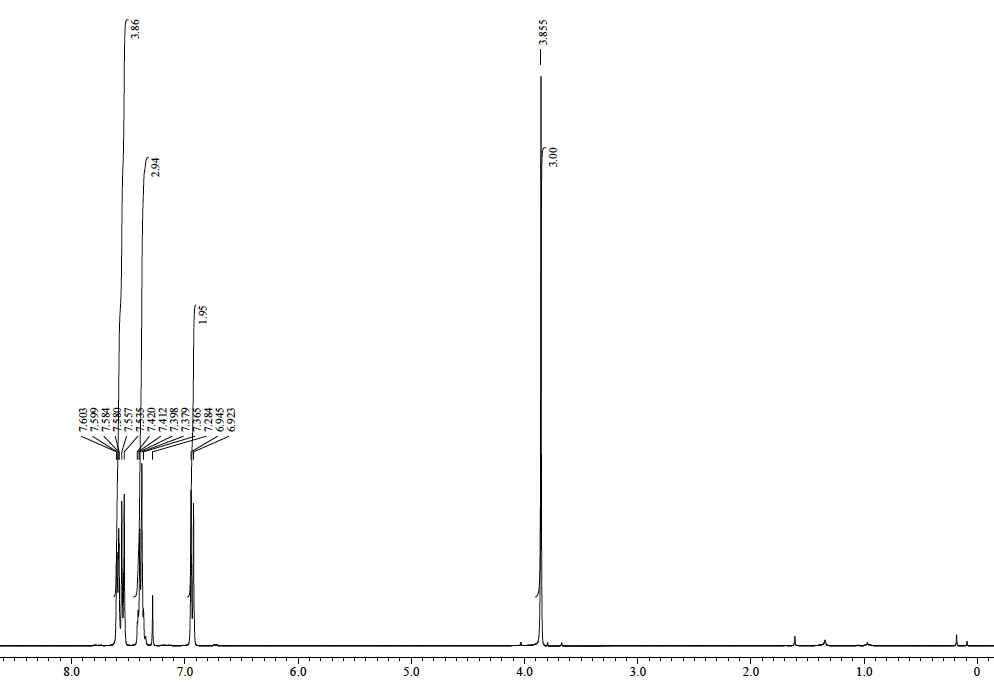


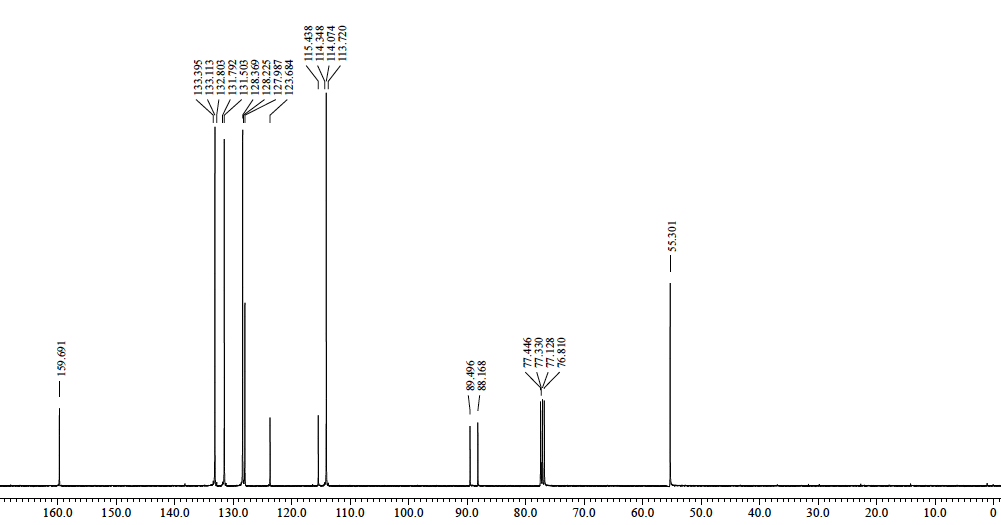


**^1^H** and **^13^C NMR** spectra of 1-methoxy-4-(phenylethynyl)benzene


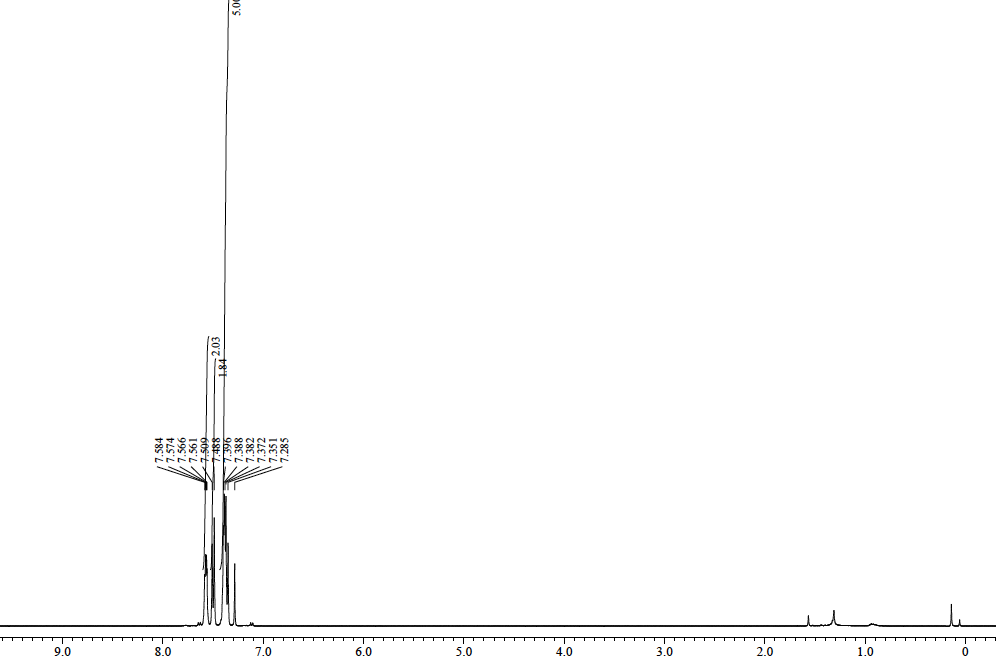


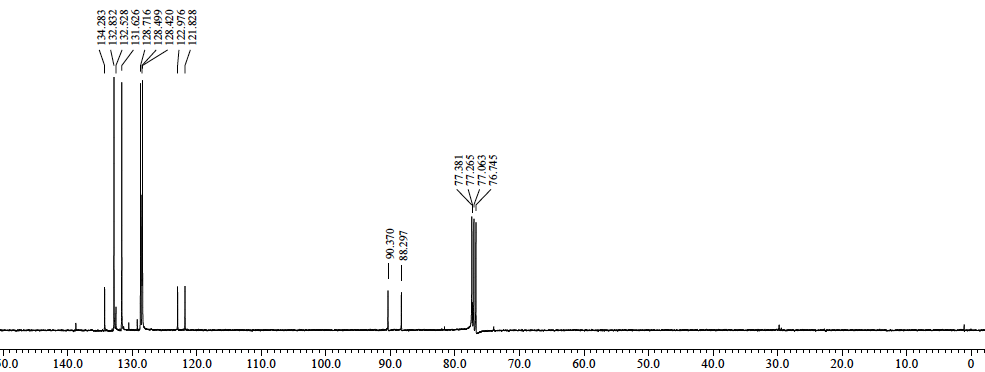


**^1^H** and **^13^C NMR** spectra of 1-chloro-4-(phenylethynyl)benzene


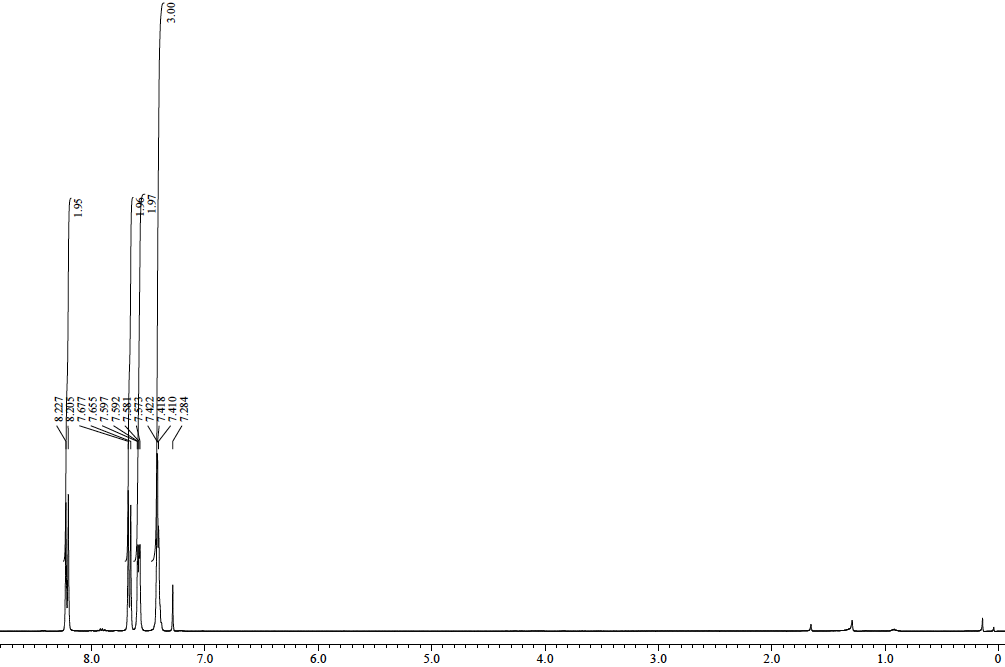


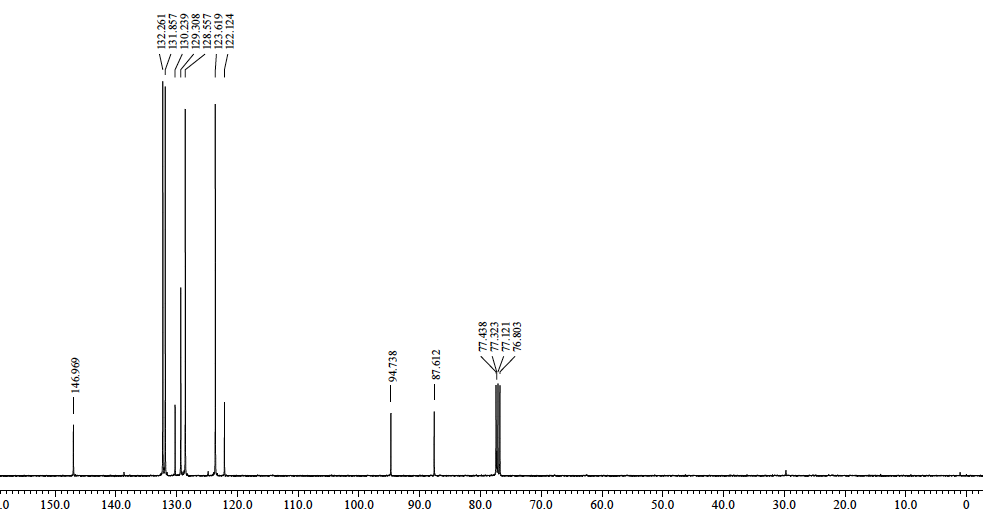


**^1^H** and **^13^C NMR** spectra of 1-nitro-4-(phenylethynyl)benzene


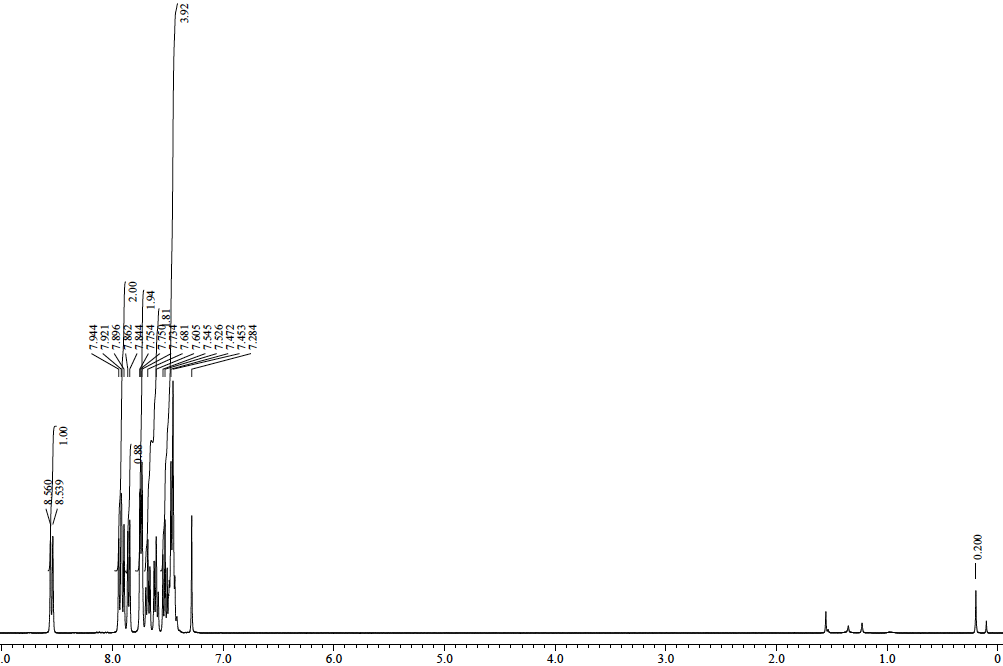


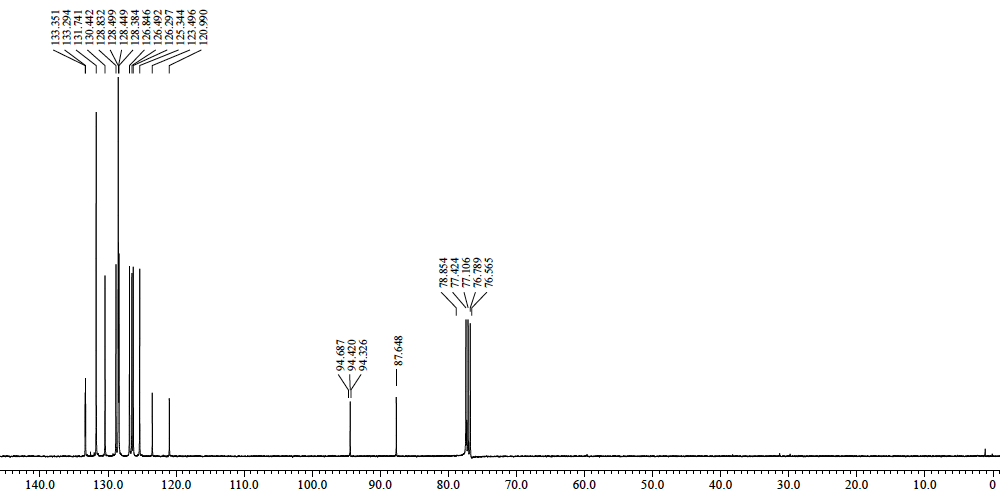


**^1^H** and **^13^C NMR** spectra of 1-(phenylethynyl)naphthalene
